# Comparative transcriptome analysis of cultivated and wild seeds of *Salvia hispanica* (chia)

**DOI:** 10.1038/s41598-019-45895-5

**Published:** 2019-07-05

**Authors:** Pablo Peláez, Domancar Orona-Tamayo, Salvador Montes-Hernández, María Elena Valverde, Octavio Paredes-López, Angélica Cibrián-Jaramillo

**Affiliations:** 1 Laboratorio Nacional de Genómica para la Biodiversidad, Unidad de Genómica Avanzada del Centro de Investigación y de Estudios Avanzados del Instituto Politécnico Nacional, Irapuato, Guanajuato Mexico; 2 Departamento de Biotecnología y Bioquímica, Centro de Investigación y de Estudios Avanzados del Instituto Politécnico Nacional, Unidad Irapuato, Guanajuato, Mexico; 3 Instituto Nacional de Investigaciones Forestales Agrícolas y Pecuarias, Celaya, Guanajuato Mexico; 4 Departamento de Soluciones Tecnológicas, Centro de Innovación Aplicada en Tecnologías Competitivas CIATEC, León, Guanajuato Mexico

**Keywords:** Gene expression, Transcriptomics

## Abstract

*Salvia hispanica* (chia) constituted an important crop for pre-Columbian civilizations and is considered a superfood for its rich content of essential fatty acids and proteins. In this study, we performed the first comprehensive comparative transcriptome analysis between seeds from cultivated varieties and from accessions collected from native wild populations in Mexico. From the 69,873 annotated transcripts assembled *de novo*, enriched functional categories and pathways revealed that the lipid metabolism was one of the most activated processes. Expression changes were detected among wild and cultivated groups and among growth conditions in transcripts responsible for triacylglycerol and fatty acid synthesis and degradation. We also quantified storage protein fractions that revealed variation concerning nutraceutical proteins such as albumin and glutelin. Genetic diversity estimated with 23,641 single nucleotide polymorphisms (SNPs) revealed that most of the variation remains in the wild populations, and that a wild-type cultivated variety is genetically related to wild accessions. Additionally, we reported 202 simple sequence repeat (SSRs) markers useful for population genetic studies. Overall, we provided transcript variation that can be used for breeding programs to further develop chia varieties with enhanced nutraceutical traits and tools to explore the genetic diversity and history of this rediscovered plant.

## Introduction

Mesoamerica is considered one of the most important centers of plant domestication^1^. The presence of populations of crops and their wild relatives throughout its landscape make it an ideal place to test for native genetic variation and how this variation relates to the history of various accessions and to their local environment^2^. *Salvia hispanica*, commonly called chia, is an annual herbaceous plant native to Mexico and North Guatemala that has served as a food source for Mesoamerican populations at least since 3500 BC^3^. In Pre-Columbian civilizations such as the Mayas and the Aztecs chia was as important as corn and bean^4^. It was extensively cultivated (between 1500 and 900 BC) for consumption of its seeds as an energy source, and for medical, artistic and religious purposes^5^. With the Spanish conquest of the Aztec Empire, the cultivation of chia was almost eradicated, probably due to European interests in other Mesoamerican crops and because of its sacred symbolism^6^. Although in some regions of Mexico consumption of wild and cultivated accessions continued, there was significant loss of traditional knowledge and rapid disappearance of wild and domesticated populations^5^. Today, the nutraceutical properties of chia seeds have made it an attractive ‘superfood’, an oilseed crop recognized worldwide.

Wild and domesticated accessions of *S. hispanica* can produce viable seedlings in assisted breeding programs^7^ opening the possibility of rewilding and genome assisted breeding^8^. Currently five accessions are cultivated broadly in Mexico, with the Spotted (Pinta) variety being the most cultivated worldwide. A previous study using Random Amplified Polymorphic DNA (RAPDs)^9^ shows that diversity of the remaining *S. hispanica* wild populations is still higher than cultivated populations, perhaps due to their broad distribution including isolated mountain regions, and low outcrossing rates reported between wild and domesticated accessions. In contrast to other crops like maize, phenotypic differences between wild and domesticated chia accessions are less obvious, although domestication syndrome traits are present, including closed calyxes, increased seed size, apical dominance, decreased pubescence, different seed coat colors and patterns, flowering period uniformity, and compacted inflorescences^9^.

Chia seeds are an important source of proteins, essential fatty acids, dietary fiber, minerals and polyphenols. Today, these seeds are among the food sources with the highest omega-3 and omega-6 fatty acids content^3^. Chia oil has a high content of ω-3 alpha-linolenic (56–64%) and ω-6 linoleic (16–22%) acids. The amount of alpha-linolenic present in chia seeds was negatively correlated with the content of its fatty acid precursors such as palmitic, oleic and linoleic acids^10,11^. Chia seeds are also a source high in protein, which makes them a very attractive food source for human nutrition and health. Chia seeds present approximately 22% of protein, an amount much higher than in other crops like oat (15%), wheat (14%), corn (14%), barley (9%) and rice (8%)^3,12^. The globulins fraction (mainly 11 S and 7 S proteins) is the most abundant in chia (52%), followed by albumins (17%), glutelins (14%) and prolamins (12%)^12,13^. Storage proteins and fatty acid contents are affected by different growth conditions; elevation for example reduced palmitic, stearic, oleic and linoleic fatty acids content^10^ but the genetic component of this variation is not well understood.

Several studies have explored the genetics and the genetic variation underlying fatty acid and storage protein contents in other oilseed crops, such as soybean^14^. Fatty acid content partially depends on the interplay between fatty acid synthesis and degradation^15^, and several acyl-lipid related genes potentially responsible for altered fatty acid content have been identified in seeds^11,14,16^. For example, mutation of *rod1* (*reduced oleate desaturation 1*), an enzyme in charge of incorporating oleic acid into phosphatidylcholine for its subsequent desaturation, reduces accumulation of linoleic and linolenic acids in triacylglycerols in *Arabidopsis* seeds^11^. In soybean mature seed transcriptomes, storage protein genes like beta-conglycinins (7 S globulin), oleosins and glycinins (11 S globulin) had higher expression with respect to all other transcripts^17,18^. During the dry seed stage, several late embryogenesis abundant (LEA) proteins and dehydrins were highly expressed due to desiccation^18^. Other genome-wide sequencing studies that assess nutraceutical properties and genetic diversity of *Salvia hispanica* have been scarce. Transcriptomes from different stages of seed development of one chia inbred line were previously generated to annotate genes of the triacylglycerol synthesis pathway and determine their expression patterns during seed development^19^.

In this study we report sixteen transcriptomes generated from seeds of eight different cultivated and wild accessions of *S. hispanica* representative of the biodiversity of this crop, with the aim to provide insights on the genetic basis of oil and protein content, and to generate novel genomic resources from accessions of various origins. We detected differences in transcript accumulation in genes involved in lipid metabolism between wild and cultivated groups. Also, by growing the same variety in different agricultural environments, we provide evidence that the expression of transcripts related to the lipid metabolism in chia seeds could be affected by different growth conditions. Genetic diversity analyses using SNPs showed that a wild accession from the state of Michoacán is more closely related to the domesticated accessions. In addition, we measured protein content between accessions through the quantification of the globulin, albumin, glutelin and prolamin fractions. Finally, due to the importance of characterizing the biodiversity of chia, we provide SSRs markers for better distinction and labelling of accessions. The transcriptomes of cultivated and wild accessions of *S. hispanica* represent a solid basis for future comparative and functional studies in this nutritious plant.

## Results

### Transcriptome sequencing, *de novo* assembly and annotation statistics

**Figure 1:**
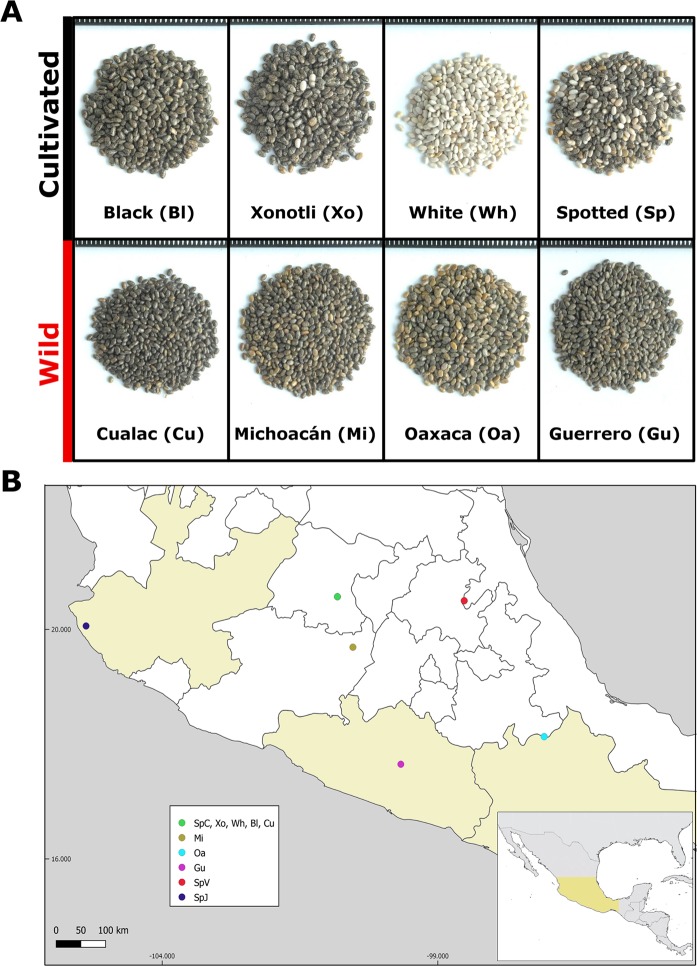
Cultivated and wild S. hispanica seeds. (**A**) Chia seeds of cultivated (Bl, Xo, Wh and Sp) and wild (Cu, Mi, Oa and Gu) accessions. A millimeter ruler (white lines in black background) was used as a scale. (**B**) Geographic location of collected wild (colored states) and cultivated (uncolored states) seeds. The three growth locations of the Spotted variety corresponded to Celaya (SpC), Jalisco (SpJ) and Veracruz (SpV).

Transcriptome libraries were made from chia seeds of four cultivated and four wild accessions. Although the Cualac variety is widely cultivated in Mexico and is considered locally semi-domesticated, in this study we placed it with the wild accessions because of its phenotypic characteristics such as small seed size and open calyxes (Fig. 1). In total, 514, 282, 900 raw reads were obtained from the sequencing of 16 libraries (Table 1). Filtering of adaptor and low-quality sequences resulted in 237,851,667 high-quality paired reads. Each library yielded at least eleven million cleaned paired reads. A reference transcriptome was *de novo* assembled using paired high-quality cleaned reads from all accessions. A total of 146,951 transcripts were assembled (116,967 with 95% of clustering similarity) with a median contig length and N50 of 687 and 1,949 base pairs (bp), respectively (Supplementary Table S1). The total size of the reference transcriptome was 171 Mb. Of the 248 conserved eukaryotic genes used to evaluate the transcriptome assembly, 244 were found in their complete state. The shortest contig was 201 bp, whereas the longest assembled contig was 66,772 bp in length. The length distribution of transcripts showed that 58,233 transcripts with a length longer than 1,000 bp were assembled (Supplementary Fig. S1). The GC content of the transcriptome was 45.5% with a peak in its sequence distribution at 43% (Supplementary Fig. S1), very similar to a previous study^19^.

**Table 1:** Summary of chia sequencing data and trimming. Paired reads left after removing adapter and low-quality sequences.

Sample ID	Accession (Chia)	Raw reads	Read bases	Input read pairs	Both surviving
Xo	Xonotli_Celaya	40,205,370	4,060,742,370	20,102,685	18,772,979 (93.39%)
Wh	White_Celaya	33,522,904	3,385,813,304	16,761,452	15,605,438 (93.10%)
Bl	Black_Celaya	30,890,870	3,119,977,870	15,445,435	14,500,700 (93.88%)
Mi	Michoacán	28,429,698	2,871,399,498	14,214,849	13,363,075 (94.01%)
Oa	Oaxaca	35,575,774	3,593,153,174	17,787,887	16,482,549 (92.66%)
Gu	Guerrero	30,713,090	3,102,022,090	15,356,545	14,277,720 (92.97%)
Cu1	Cualac_Celaya	35,433,590	3,578,792,590	17,716,795	15,984,930 (90.22%)
Cu2	Cualac_Celaya	24,941,072	2,519,048,272	12,470,536	11,116,161 (89.14%)
SpC1	Spotted_Celaya	37,127,126	3,749,839,726	18,563,563	17,428,851 (93.89%)
SpC2	Spotted_Celaya	23,993,832	2,423,377,032	11,996,916	11,012,809 (91.80%)
SpC3	Spotted_Celaya	27,924,766	2,820,401,366	13,962,383	12,816,006 (91.79%)
SpV1	Spotted_Veracruz	30,786,878	3,109,474,678	15,393,439	14,000,447 (90.95%)
SpV2	Spotted_Veracruz	32,944,202	3,327,364,402	16,472,101	15,503,037 (94.12%)
SpV3	Spotted_Veracruz	30,570,926	3,087,663,526	15,285,463	13,837,446 (90.53%)
SpJ1	Spotted_Jalisco	32,106,074	3,242,713,474	16,053,037	14,967,600 (93.24%)
SpJ2	Spotted_Jalisco	39,116,728	3,950,789,528	19,558,364	18,181,919 (92.96%)

To assess transcripts annotations, the reference transcriptome was mapped to the UniProt Plant Protein database. A total of 69,873 transcripts were mapped, representing 12,583 unique identifiers and more than 10,000 different gene products (Supplementary Table S2). E-value distribution showed that 73% of the alignments had an E-value less than E-25 (Supplementary Fig. S1). Two species contributed with the greatest number of best hits of the aligned transcripts. Eighty percent of the best hits corresponded to *Arabidopsis thaliana* and ten percent to *Oryza sativa* subsp*. japonica* (Supplementary Fig. S1). As previously observed in other studies, small transcripts were poorly annotated; however, 73.6% of the transcripts longer than 1,000 bp were annotated (Supplementary Fig. S1). Other non-protein databases would increase the number of annotated transcripts. In terms of abundance, 56,136 transcripts had a value greater or equal to 0.5 transcripts per million reads (TPM) on average between the accessions (Supplementary Fig. S1). We further subjected the transcripts to an enrichment analysis based on the Gene Ontology (GO) and KEGG pathways terms (Supplementary Fig. S1; Supplementary Table S3). Within the molecular function category, binding, catalytic activity and protein binding were among the most highly represented categories. Cellular process, cellular metabolic process and metabolic process were the top three represented categories in the biological process classification. Among the cellular component category, organelle part, cell part and cell were major GO terms. For KEEG categories, the most highly represented were metabolic pathways, biosynthesis of secondary metabolites and carbon metabolism. Within the categories related to metabolism, it is worth mentioning that glycerophospholipid, glycerolipid and fatty acid metabolisms were evident (Supplementary Fig. S1).

In soybean seeds at the dry seed stage, RNA sequencing (RNA-seq) profiling exhibited high expression of transcripts annotated as late embryogenesis abundant (LEA) proteins and dehydrins, which help to stabilize and preserve membranes, nutrients and proteins in low water conditions^18^. We also found that *LEA* and storage proteins were among the most abundant transcripts (Supplementary Table S4). Within the top 10 most abundant transcripts, a protein likely involved in the acquisition of desiccation tolerance (glucose and ribitol dehydrogenase) and a vicilin-like antimicrobial peptide were detected (Table 2). Most of the different transcripts assigned to a particular gene product showed signals of expression in all the accessions (Supplementary Fig. S2). The gene products that were only detected in either wild or cultivated accessions were few, 13 and 18, respectively. Interestingly, the RNA-dependent RNA polymerase 1 (RDR1), which is involved in antiviral silencing and wax biosynthesis^20^, was detected only in the cultivated accessions.

**Table 2:** Top 10 most expressed annotated transcripts. Expression percentage was determined based on the transcripts per million values from all accessions.

Protein Name	Uniprot Acc	E-value	Expression (%)
18 kDa seed maturation protein	Q01417	1.00E-16	50984
Protein LE25	Q00747	3.00E-19	39448
11 S globulin subunit beta	P13744	3.00E-132	28687
Protein SLE3	C6T0L2	7.00E-45	26749
Vicilin-like antimicrobial peptides 2–2	Q9SPL4	3.00E-122	26600
2 S albumin	Q39649	3.00E-06	24034
Late embryogenesis abundant protein 31	Q9LJ97	5.00E-55	6528
Late embryogenesis abundant protein D-34	P09444	8.00E-72	6420
Protein SLE1	I1N2Z5	3.00E-44	6296
Glucose and ribitol dehydrogenase	Q5KTS5	6.00E-165	5617

### Identification and expression analyses of lipid-related genes

Due to the nutritional interest in chia seeds for its high oil content, lipid-related genes were identified and classified using the *Arabidopsis* Acyl-Lipid (ARALIP) database among the different accessions. Transcripts homologous to a total of 683 loci from *Arabidopsis* that encode genes related to acyl lipid metabolic and signaling pathways were identified in the reference transcriptome (Supplementary Table S5). The Spotted cultivated variety, one of the accessions with the highest sequencing depth, presented the highest number of homologous genes from *Arabidopsis* related to lipid metabolism (Supplementary Fig. S3). The category with the highest number of genes identified was fatty acid elongation and wax biosynthesis. However, taking account the background input in the database, other categories like phospholipid signaling (90%), triacylglicerol biosynthesis (74%), eukaryotic phospholipid synthesis and editing (92%) and fatty acid synthesis (89%) were better represented. In terms of presence or absence of gene models, the biggest difference between accessions was observed in the phospholipid signaling category. A total of 541 genes were shared between accessions and 563 genes were detected in all the cultivated accessions, while 548 were present in all wild seeds (Supplementary Fig. S4).

**Figure 2:**
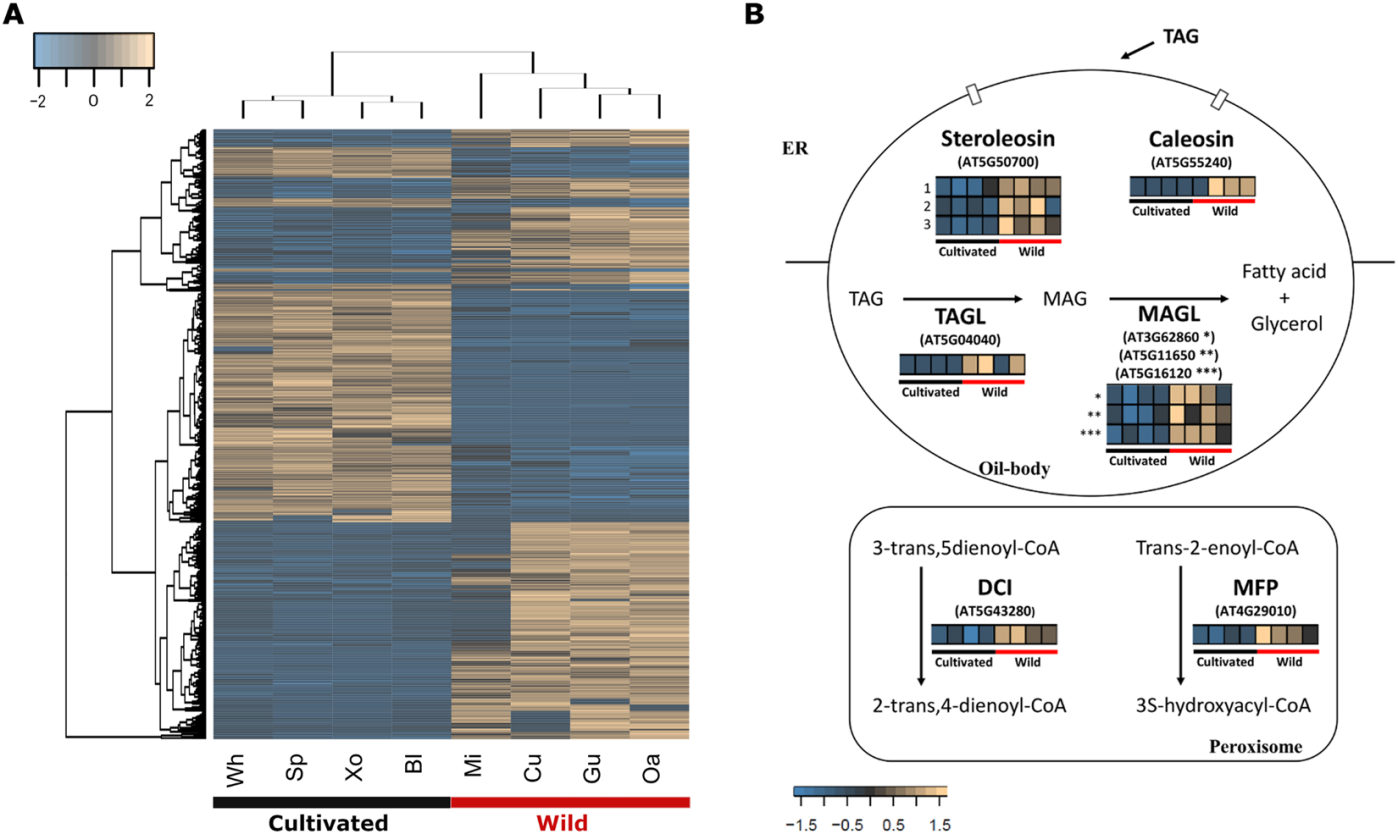
Differentially expressed transcripts among cultivated and wild accessions. (**A**) Heatmap showing the expression of 1,034 significantly (FDR 5%) differentially expressed transcripts in cultivated (black line) and wild (red line) seeds of *S. hispanica*. (**B**) Schematic representation of acyl-related proteins involved in triacylglycerol and fatty acid degradation containing expression based heatmaps of their identified transcripts in wild and cultivated accessions. Three transcript isoforms were identified for the steroleosin protein (numbers on the left side of the heatmap). Scale bars represent the degree of gene expression. Abbreviations: TAGL, triacylglycerol lipase; MAGL, monoacylglycerol lipase; DCI, 3,5-delta2,4-dienoyl-CoA isomerase; MFP, multifunctional protein; Xo, Xonotli; Wh, White; Bl, Black; Mi, Michoacán; Oa, Oaxaca; Gu, Guerrero; Cu, Cualac; Sp, Spotted.

**Figure 3:**
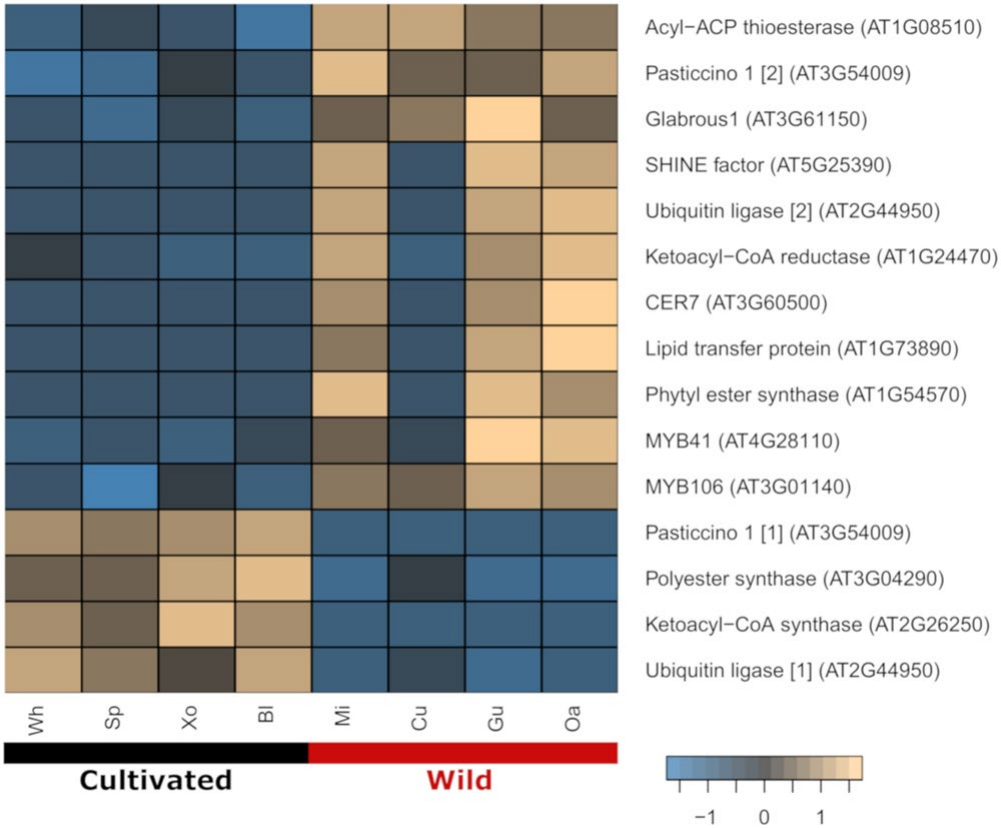
Differentially expressed transcripts associated to fatty acid elongation and wax biosynthesis between cultivated and wild accessions. Heatmap of 15 significantly (FDR 5%) differentially expressed transcripts in cultivated (black line) and wild (red line) seeds of chia. Transcript isoforms corresponding to two gene models (AT2G44950 and AT3G54009) are shown (numbers in brackets). Scale bar represents the degree of expression.

To determine if acyl-lipid related transcripts exhibited expression polymorphisms between cultivated and wild accession groups, variation in their expression levels were evaluated. A total of 1,034 assembled transcripts showed differences in their expression levels (Fig. 2A), with 474 up-regulated and 560 down-regulated in cultivated varieties compared to wild accessions; 647 were annotated with the Uniprot database and 121, representing 76 gene models, were recognized as acyl-lipid related transcripts (Supplementary Table S6). The top six gene ontology categories of the 647 transcripts were cellular process, binding, cellular metabolic process, metabolic process, organic substance metabolic process and primary metabolic process. Most of the acyl-lipid related transcripts (65 transcripts) were upregulated in the cultivated group. We found that very long-chain fatty acid metabolic process, fatty acid biosynthetic process and fatty acid metabolic process were the top three gene ontology categories for lipid-related transcripts, which highlights differences in the expression of genes involved in the synthesis of different types of lipids among cultivated and wild accessions. We detected that a transcript annotated as *ROD1*, which promotes desaturation of oleic acid into linoleic and linolenic acids^11^, was up-regulated in the wild group. Interestingly, all the differentially expressed transcripts involved in triacylglycerol and fatty acid degradation were up-regulated in the wild group (Fig. 2B). Three transcript isoforms encoding steroleosin and one encoding caleosin, two binding oil-body surface proteins associated with synthesis and degradation of triacylglycerols^21^, were accumulated in the wild pool. We detected greater expression of one triacylglycerol and three monoacylglycerol lipase transcripts in the wild accessions involved in lipid breakdown and associated with oil bodies. Also, *DCI* (3,5-delta2,4-dienoyl-CoA isomerase) and *MFP* (multifunctional protein), two transcripts encoding two important proteins of the fatty acid β-oxidation process^21^, found in peroxisomes, were up-regulated (Fig. 2B). Since we detected signals of transcription of *RDR1* only in cultivated accessions, we looked for differentially expressed transcripts involved in fatty acid elongation and wax biosynthesis^20^. We found that most of the transcripts involved in wax biosynthesis were up-regulated in the wild group, including the transcript of the ribonuclease *CER7*, which regulates the transcription of *CER3*, a gene required for wax biosynthesis (Fig. 3). Based on the expression of genes that regulate wax biosynthesis in both groups, we hypothesized a reduction of wax biosynthesis in cultivated accessions through an active RDR1-mediated silencing of *CER3*^20^.

**Figure 4:**
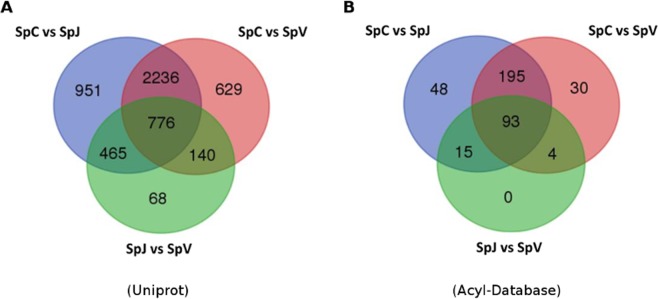
Venn diagrams representing the number of genes from the Uniprot (**A**) or Acyl (**B**) databases differentially expressed for each of the three environmental comparisons of the Spotted variety.

Fatty acids and storage proteins contents in plants are also a result of the interaction of the genotype and the environment^14^. We were interested in the identification of transcripts, mainly in those involved in lipid metabolism, that changed expression in relation to their place of cultivation. Plants from the Spotted variety were grown in the states of Guanajuato (Celaya), Veracruz and Jalisco, which have similar latitude but differ in local environmental factors such as elevation, rainfall, and annual average temperature (Table 1). Expression analysis was evaluated between any two of the three locations for the Spotted variety. A total of 10,730 transcripts annotated with the Uniprot database (Supplementary Table S7) were found differentially expressed for the three sites. Comparison between seeds from Celaya and Jalisco yielded 8,131 transcripts differentially expressed, the greatest amount of the three comparisons. Seeds grown in Veracruz and Jalisco had a lower number of differentially expressed transcripts (1738). A total of 5,265 protein transcripts were differentially expressed across locations (Fig. 4A). Seven hundred seventy- six were differentially expressed in the three comparisons. Cellular process, regulation of biological process, cellular metabolic process, biological regulation, regulation of cellular process and metabolic process were the top six represented gene ontology categories for these transcripts. Differential expression analysis of transcripts related to lipid metabolism yielded 2,243 transcripts corresponding to 385 different gene models differentially expressed in the three locations (Supplementary Table S8). The Spotted seeds from Jalisco and Veracruz had a lower number of differentially expressed transcripts. A Venn diagram of differentially expressed genes related to lipid metabolism indicated that 93 genes were differentially expressed in the three comparisons (Fig. 4B). Of these genes, eighteen transcripts corresponded to the fatty acid elongation and wax biosynthesis category. Other categories with the greatest number of transcripts were triacylglycerol biosynthesis (15) and triacylglycerol and fatty acid degradation (11). Among the differentially expressed genes, members of lipid-related pathways and regulators such as the mitochondrial beta-ketoacyl-acp synthase, the ketoacyl-acp reductase, a monoacylglycerol lipase, an abscisic acid insensitive transcription factor, a triacylglycerol lipase, an auxin response factor and an AP2/EREBP transcription factor were detected. In summary, transcripts related to fatty acid elongation, wax biosynthesis, triacylglicerol synthesis and degradation were highly represented in the transcriptomes and several of them were differentially expressed in the different growth locations and between wild and cultivated accessions, suggesting important roles of these processes in the lipid metabolism of chia seeds and the diversification of chia.

### Sequence polymorphism identification and genetic diversity analysis

**Figure 5:**
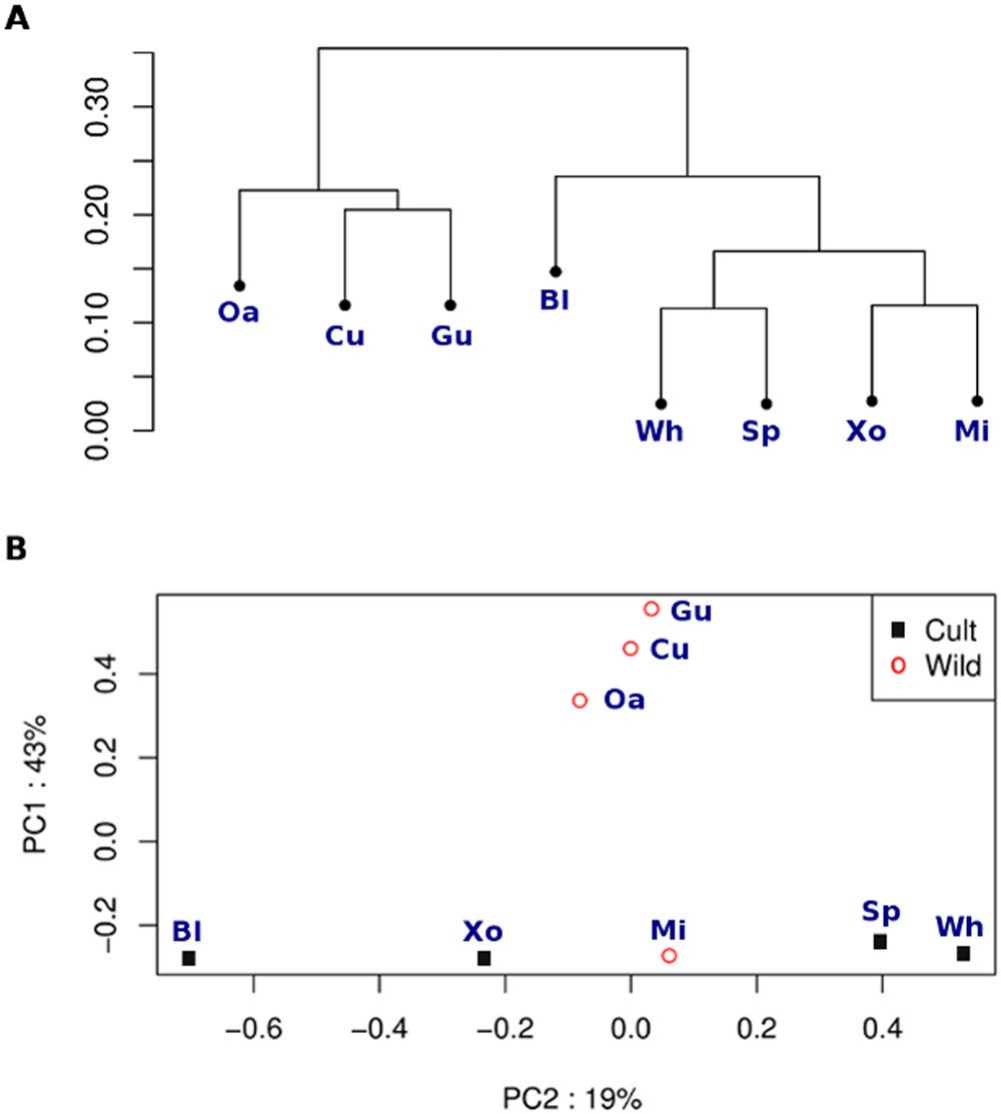
Genetic variation in wild and cultivated seeds of *S. hispanica*. Dendrogram (**A**) and principal component analysis plot (**B**) based on SNPs.

Genetic diversity in wild accessions of chia is higher than in commercial domesticated accessions, which has led to the hypothesis that chia was domesticated only once^9^. To get an insight into the genetic diversity and relationships between *S. hispanica* accessions, a total of 23,641 high-quality genome-wide SNPs were identified. Genetic similarity among accessions was studied by exploring the number of shared alleles between accessions through an Identity By State (IBS) analysis, represented by a dendrogram, that resulted in two main clusters (Fig. 5A). One cluster was composed of the three wild accessions from Guerrero, Oaxaca and the Cualac accession. Cualac is considered a commercial variety and is cultivated in Mexico with a considerable seed yield per hectare, yet our results together with evidence of its wild phenotypic characteristics such as small seed size and open calyxes, suggest that Cualac plants are wild relatives. In the other cluster, the wild accession from Michoacán clustered together with the cultivated varieties. Accessions from this area (The Trans-Mexican Volcanic Belt) have shown close genetic relatedness with cultivated varieties, which makes them good candidates to be the direct wild relatives of the domesticated accessions^9^.

Principal component analysis (PCA) using the identified SNPs revealed that the two first components accounted for 62% of the total variation (Fig. 5B). Consistent with previous observations, cultivated accessions were clustered more closely together than wild accessions. Accessions such as Black, White, Spotted and Xonotli were situated together along the x-axis with respect to the principal component explaining the largest variation. In contrast, wild accessions were more dispersed. As expected, the accession from Michoacán is strongly separated from the rest of the wild accessions and is closer to the cultivated ones. However, a considerable distance was observed among the remaining wild accessions (Gu, Oa and Cu). Overall, these results support previous evidence of a higher genetic diversity in wild populations and helped categorize cultivated accessions, including their closest wild relatives.

### Storage proteins profiles

Storage proteins accumulate significantly in seeds to provide the required nutrients during germination and seedling growth. To our knowledge, differences regarding the protein content of storage proteins between Mexican chia accessions are evaluated for the first time here. We included the comparison of protein fraction content between accessions classified as commercial accessions (Xo, Wh, Bl, Cu and Sp), with the Spotted variety grown in different locations. We evaluated four essential seed storage proteins, albumin, globulin, prolamin and glutelin. The overall pattern of accumulation of these storage proteins in chia seeds showed that globulins are the most abundant proteins overall, followed in order by albumins, glutelins and prolamins, as previously reported for chia^12^ (Table 3). Significant (p ≤ 0.05) differences were detected between protein concentrations among the different accessions for the albumin and glutelin fractions. However, no significant difference was detected for the most (globulins) and the least (prolamins) abundant proteins between any of the accessions evaluated. For the accessions grown in a single location, Celaya (Wh, Xo, Cu, Bl and SpC), we found that the Black variety significantly accumulates higher amounts of albumins compared with the rest of the accessions (Table 3). The content of glutelins was significantly higher in the White, Xonotli and Cualac accessions. The content of seed storage proteins measured for the accessions corresponding to the Spotted variety grown in three different locations indicates that albumin is the only protein that significantly varies in these accessions and that its concentrations could be more affected by growth conditions.

**Table 3:** Storage protein fractions of wild and cultivated chia seeds. Data was represented as means ± SE of the fractions (%). Mean values sharing the same letter corresponding to a particular protein quantification were not significantly different (p ≤ 0.05).

Variety	Storage Protein Concentration (% fraction)			
	Albumin	Globulin	Prolamin	Glutelin
White (Wh)	20.8 ± 1.2^ab^	53.4 ± 5.9^a^	10.7 ± 0.4^a^	15.1 ± 1.4^bc^
Xonotli (Xo)	20.1 ± 0.7^ab^	53.1 ± 0.4^a^	10.3 ± 0.1^a^	16.5 ± 0.5^bc^
Cualac (Cu)	21.4 ± 0.5^ab^	51.9 ± 3.5^a^	9.8 ± 0.6^a^	16.9 ± 0.4^c^
Black (Bl)	28.5 ± 2.1^c^	50.0 ± 0.5^a^	10.5 ± 0.8^a^	11.0 ± 0.1^a^
Spotted_Celaya (SpC)	21.8 ± 0.3^ab^	54.7 ± 1.4^a^	10.4 ± 0.4^a^	13.1 ± 0.6^ab^
Spotted_Jalisco (SpJ)	23.3 ± 1.7^b^	52.7 ± 0.9^a^	10.0 ± 0.1^a^	13.9 ± 1.3^abc^
Spotted_Veracruz (SpV)	18.9 ± 0.2^a^	56.8 ± 0.6^a^	11.1 ± 0.9^a^	13.1 ± 2.0^ab^

### Identification of Simple Sequence Repeats

Simple sequence repeats (SSRs), or microsatellites, are widely employed for genotyping and can be used to infer genetic relations and as tools to map quantitative trait loci^22^. To provide a valuable tool for a better genotypification of *S. hispanica*’s accessions, we identified SSRs from the transcriptomes. A total of 24,402 SRRs of eight different type of motifs (2 to 9) were identified in 19,789 transcripts (13.4% of the reference transcriptome). The top five most abundant SSRs were dinucleotide motifs (78.6%), then trinucleotide (19.1%), hexanucleotide (0.85%), tetranucleotide (0.83%) and lastly pentanucleotide (0.45%). The abundance of SSRs tends to decrease as the length increases. The most abundant motif was the dinucleotide motif AG, comprising 13.9% of the motifs, whereas the abundance of the most abundant trinucleotide motif GAT was just 0.95%. Moreover, to characterize SSRs, we designed primers for amplification of SSR loci that were detected in the transcriptomes of all the accessions. We provide a total of 202 different primer pairs for SSR loci of trinucleotide motifs (137), tetranucleotide (43), pentanucleotide (14) and hexanucleotide (8; Supplementary Table S9). Motif repetitions of selected SSRs loci vary from 5 to 13 repetitions, and the length of SSRs ranged from 15 to 42 nucleotides.

## Discussion

There is a growing need for the genetic characterization of *S. hispanica* accessions to understand the genetic basis controlling various of its nutraceutical traits. Transcriptome-based studies are advancing our understanding of gene expression patterns associated with valuable plant traits from crop accessions and their wild relatives^23^. Understanding the genetic basis of variation in desired crop phenotypes is also essential for better implementation of plant breeding programs and plant genetic diversity conservation. *Salvia hispanica* is an understudied and until recently underutilized oilseed crop with an ancient history of human interaction. Plants from its native Mexico contain unexplored genetic diversity and a nutritional and therapeutic potential yet to be discovered. In this work we sequenced seed transcriptomes of eight different wild plants and cultivated accessions of chia from Mexico, generating an important genomic resource for understanding the native biodiversity of the crop, in particular the identification of sequence and expression polymorphisms of transcripts related to lipid metabolism.

Overall, the number of reads and the number of chia accessions sequenced in this study allowed the assembly of the most diverse and complete reference transcriptome for the species to date. We obtained insights of the predominant transcripts in chia mature seeds and identified genes involved in metabolic pathways controlling lipid metabolism, and particularly fatty acid content, which is among the most valuable traits in oilseed crops. We were also able to provide more than seven thousand gene products shared by all the samples, which constitute the expression landscape of this species. The identification of *LEA* and responsive to dehydration transcripts as part of the most abundant transcripts (Table 2 and Supplementary Table S4) confirmed the seed stage of the samples and was consistent with previous reports in soybean^18^. The enriched functional categories and statistically enriched pathways related to the lipid metabolism supports the importance of lipids in chia seeds at a mature stage. As with younger development stages in chia seeds, transcripts related to the glycerophospholipid metabolism were the most abundant transcripts related to lipid metabolism^19^.

An important characteristic that distinguishes cultivated from wild chia seeds is seed size. During late stages of seed development triacylglycerols accumulate in the oil bodies of embryos, which is correlated with seed size^24^. Domestication of chia could therefore involve the accumulation of storage lipids in cultivated accessions. We found that genes responsible for the biosynthesis and degradation of triacylglycerols and fatty acids have differences in expression between cultivated and wild accessions. In particular, up-regulated genes involved in lipid breakdown in wild accessions could be direct targets of selection to improve oil content in seeds^15^. Expression changes in these transcripts suggest that wild accessions have an active fatty acid breakdown that reduces accumulation of triacylglycerols. Modifications in fatty acid breakdown could lead to a greater accumulation of triacyglycerols and changes in fatty acids content^24,25^. In addition, it is also known that active fatty acid degradation could imply an active fatty acid biosynthesis^15^. We also found increased expression of transcripts promoting fatty acid biosynthesis in wild accessions like *ROD1*. The important roles of fatty acid degradation in germination or in stress responses makes this process an interesting target of study in future chia research. Cultivated varieties may present more oil content than wild varieties but with different fatty acid composition.

In addition to lipid breakdown transcripts, it was intriguing that several transcripts involved in wax biosynthesis were differentially expressed between wild and cultivated groups and that we did not detect signals of transcription of *RDR1* in wild accessions. Differences in the expression of genes associated with wax accumulation have been correlated with aerial phenotypic differences among wild and cultivated accessions of tomato^26^. The wild tomato *Solanum pennellii* is adapted to a desert climate and, compared to cultivated tomatoes, has a cuticle with greater amounts of waxes that limits water loss. The cuticle consists of a waxy layer that surrounds the embryo in seeds and serves as protection against biotic and abiotic stresses, as well as contributes also to maintain their dormant state. Our results suggest that there are differences in wax deposition between chia seeds of cultivated and wild accessions. Future studies may validate this phenotypic difference in chia, the role of wax biosynthesis in this plant and explore if it corresponds to a domestication footprint.

Previous evidence showed that in chia growth conditions affect oil content^10^, but the transcriptional basis for those differences is unknown. Although differences in expression in Spotted accessions grown in different environments was not a surprise, the identity of those transcripts represents valuable information for understanding and eventually modifying lipid metabolism regulation in chia. Very interesting transcripts related to the lipid metabolism were differentially expressed across sites in the Mexican landscape. One of those transcripts was an *AP2/EREBP* transcription factor (DN38422). This transcript was overexpressed in the Spotted accessions from Jalisco. AP2/EREBP transcription factors regulate the expression of genes related to fatty acid synthesis^27^. The member identified in chia regulates cuticular wax biosynthesis, which begins with the synthesis of 16:0 and 18:0 fatty acids. The AP2/EREBP transcription factors and cuticular waxes have very important roles during stress abiotic responses induced by light, temperature or elevation, just to mention a few examples^28^. Overexpression of members of the *AP2/EREBP* family in other plants resulted in increased levels of essential fatty acids like palmitic and linolenic acid^29^, making this family very attractive for further studies in chia.

Despite the resurgence of chia as a valuable oilseed crop, there is almost no information regarding its neutral standing genetic diversity from its place of origin. The only other study exploring genetic diversity among accessions of chia from Mexico^9^ concluded that the genetic diversity in wild accessions was higher compared to domesticated ones and that low diversity of domesticated accessions suggests a single origin of domestication. Our results, using SNPs, also showed a loss of diversity in cultivated accessions. We find that the wild accession from Michoacán was more closely related to cultivated accessions than those of Guerrero and Oaxaca, supporting Cahill’s observation that accessions from this area are likely to be the wild chia relatives^9^. We hope that our marker development and identification of changes in gene expression among cultivated and wild accessions will facilitate further studies in chia’s origin and genetic diversity.

Protein content, especially derived from storage proteins, together with oil content constitutes a relevant nutraceutical characteristic to be studied in chia. We included a profile using mainly cultivated accessions, of four abundant storage proteins that could be important as a source of dietary protein. In general, our results suggest that the content of albumin, globulin, prolamin and glutelin among the accessions is very similar. This could mean that all accessions produce seeds that could serve as excellent protein sources with high quality protein that may fulfill protein needs in human nutrition. Importantly, although the Cualac variety presents wild-related phenotypic characteristics such as small seed size, its storage protein content was in most cases not significantly different with respect to the other accessions.

The transcriptome analysis of seeds from cultivated and wild accessions of *S. hispanica* contributes to the identification and characterization of transcript variation in this rediscovered nutritious plant with an almost forgotten history. Information and data generated in this study will be valuable for further understanding differences in fatty acids and protein content among seeds of cultivated and wild accessions belonging to the same species. The data reported here will help this initiative and further genomic projects to expand our knowledge of this valuable Mesoamerican crop.

## Methods

### Plant material

All *Salvia hispanica* L. varieties (Xonotli, Spotted (Pinta), Cualac, White (Blanca) and Black (Negra)) were grown in the experimental field of the Instituto Nacional de Investigaciones Forestales, Agrícolas y Pecuarias (INIFAP) located in Celaya, Guanajuato, Mexico (20.578°, −100.822°). In addition, *S. hispanica* L. var. Spotted was also grown in Tomatlán (Jalisco; 20.067°, −105.3855°) and Huayococotla (Veracruz; 20.508°, −98.528°) to address a possible environmental contribution to gene expression profiles. Seeds were collected at a desiccation stage and were stored at room temperature until RNA extraction (180 days after flowering). Some wild *S. hispanica* plant seeds were collected in the municipalities of Teotitlan de Flores Magón (Oaxaca), Ciudad Hidalgo (Michoacán) and Leonardo Bravo (Guerrero). These seeds were approximately two times older than the rest.

### RNA extraction, Library Preparation and Sequencing

Total RNA was extracted as described by Wang *et al*.^30^ for isolation of RNA from cereal seeds. The 16 transcriptome libraries were constructed using the TruSeq RNA Library Preparation Kit (v2) and paired-end sequenced (100 pb) on an Illumina HiSeq 4000 sequencing system (Macrogen, Inc.). All sequence data are available at the SRA database (SRP137146).

### Assembly, annotation and sequence data analyses

Raw sequences were trimmed to discard low quality and adapter sequences using Trimmomatic (version 0.32)^31^. A reference transcriptome was *de novo* assembled with Trinity version 2.1.1 using paired high-quality cleaned reads from 8 libraries (Xo, Wh, Bl, Mi, Oa, Gu, Cu1 and SpC1), one for each accession^32^. The quality of the assembly was evaluated aligning reads back to the reference transcriptome and the completeness of conserved eukaryotic genes. Transcripts were clustered with CD-HIT at 95% of identity^33^. Transcripts were mapped against the UniProt Plant Protein database (release 2016_09) using BLASTX (E-value ≤ 1.0E-5). Abundance estimation of transcripts was determined with RSEM software^34^. Only the best hit for each transcript was considered for E-value and species distribution. KOBAS version 3.0 was used with default parameters for Gene Ontology and KEGG pathways enrichment analyses^35^. Five libraries (Cu1, Cu2, Mi, Oa and Gu) of wild accessions and four of cultivated varieties (Xo, Wh, Bl and SpC1) were used to detect presence or absence of gene products. For expression distribution total Trinity transcripts were considered (annotated and non-annotated transcripts). For Venn diagram comparison (http://bioinformatics.psb.ugent.be/webtools/Venn/), transcripts were considered absent when RSEM expected count was zero.

### Lipid-related gene expression analyses

To identify and classify lipid related transcripts, contigs from the reference transcriptome were mapped against the ARALIP database (http://aralip.plantbiology.msu.edu/about) using BLASTX (E-value ≤ 1.0E-3). To identify differentially expressed transcripts among the cultivated (Xo, Wh, Bl, SpC1) and wild groups (Oa, Gu, Mi, Cu1) and in the Spotted variety grown in different locations (SpC1,2,3; SpV1,2,3; SpJ1,2), analysis of pairwise differential expression between locations was performed using DESeq. 2^36^. Input matrices were constructed based on RSEM expected counts. Transcripts were considered as significantly differentially expressed if FDR was less than 1% (adjusted p-value < 0.01) and 5% (adjusted p-value < 0.05) for the environmental and groups comparisons, respectively. Hierarchical clustering heatmaps were performed using the heatmap.2 function from the gplopts package (R software).

### Storage proteins profiles

Chia seeds were soaked in distilled water (1:10; w/v) for 1 h to allow mucilage production, then seeds were frozen overnight (−80 °C) and freeze-dried. The dried mucilage was mechanically separated from the seed by rubbing on a mesh^37^ and mucilage-free seeds were ground in a cold mild, and the flour passed through a 0.5 mm mesh. The flours were defatted with hexane (1:10; w/v) at 60 °C for 2 h in a Buchi E-816 SOX extraction unit (Flawil, Switzerland), and the flour was left overnight under a hood and then stored at 4 °C until use^38^. Proteins from chia flours were fractionated according to the Osborne (1924) classification and based on the method reported by Orona-Tamayo *et al*.^13^. Data was represented as means ± SE of the fractions (%). Statistical analysis for protein content comparisons was performed using the SPSS software (version 14.0) through one-way ANOVA followed by Duncan’s test (n = 3; p ≤ 0.05).

### Sequence polymorphism analysis

Cleaned sequences from the different libraries (Xo, Wh, Bl, Mi, Oa, Gu, Cu1 and SpC1) were mapped against the reference transcriptome using Bowtie 2 with default options^39^. SAMtools software package (v.1.3) and the reference transcriptome (*de novo* assembly) were used to obtain sorted BAM and mpileup files^40^. SAMtools mpileup and BCFtools (with the options for biallelic sites, SNPs only, no-BAQ, minimum mapping quality of 20 and minimum base quality of 25) were used for SNP calling. SNPs were further filtered with VCFtools if genotypes called were below 80% across all samples, minimum mean depth was below 10, minimum quality score was below 30 and minor allele count was less than 3. Distance matrix calculation, hierarchical cluster analysis, group identification and PCA analysis were carried out using the gdsfmt and SNPRelate packages^41^.

### SSR identification

Microsatellites were identified from the reference transcriptome using the microsatellite identification tool MISA with default parameters (http://pgrc.ipk-gatersleben.de/misa/). BatchPrimer3 was used for SSR primers design^42^. SSR primers were only reported if reads supporting the reference transcripts were detected in all transcriptomes.

## Supplementary information

Supplementary information

Supplementary Table S2

Supplementary Table S3

Supplementary Table S4

Supplementary Table S5

Supplementary Table S6

Supplementary Table S7

Supplementary Table S8

Supplementary Table S9

## Data Availability

The datasets generated during and/or analyzed during the current study are available in the SRA database (SRP137146).
