# Supplementary material for: Comparative transcriptome analysis of cultivated and wild seeds of Salvia hispanica (chia)
Source: Sci Rep. 2019 Jul 5;9:9761. doi: 10.1038/s41598-019-45895-5 (PMC6611817; doi:10.1038/s41598-019-45895-5)
Supplement: Supplementary file 1 — Supplementary information [file 41598_2019_45895_MOESM1_ESM.pdf]

Comparative transcriptome analysis of cultivated and wild seeds of *Salvia hispanica* (chia)

Pablo Peláez[1], Domancar Orona-Tamayo[2,4], Salvador Montes Hernández[3], María Elena Valverde[2], Octavio Paredes-López[2], Angélica Cibrián-Jaramillo[1*]

[1] Laboratorio Nacional de Genómica para la Biodiversidad, Unidad de Genómica Avanzada del Centro de Investigación y de Estudios Avanzados del Instituto Politécnico Nacional, Irapuato, Guanajuato, México.

[2] Departamento de Biotecnología y Bioquímica, Centro de Investigación y de Estudios Avanzados del Instituto Politécnico Nacional, Unidad Irapuato, Guanajuato, México.

[3] Instituto Nacional de Investigaciones Forestales Agrícolas y Pecuarias, Celaya, Guanajuato, México.

[4] Departamento de Soluciones Tecnológicas, Centro de Innovación Aplicada en Tecnologías Competitivas CIATEC-León, México.

*** Corresponding,** angelica.cibrian@cinvestav.mx

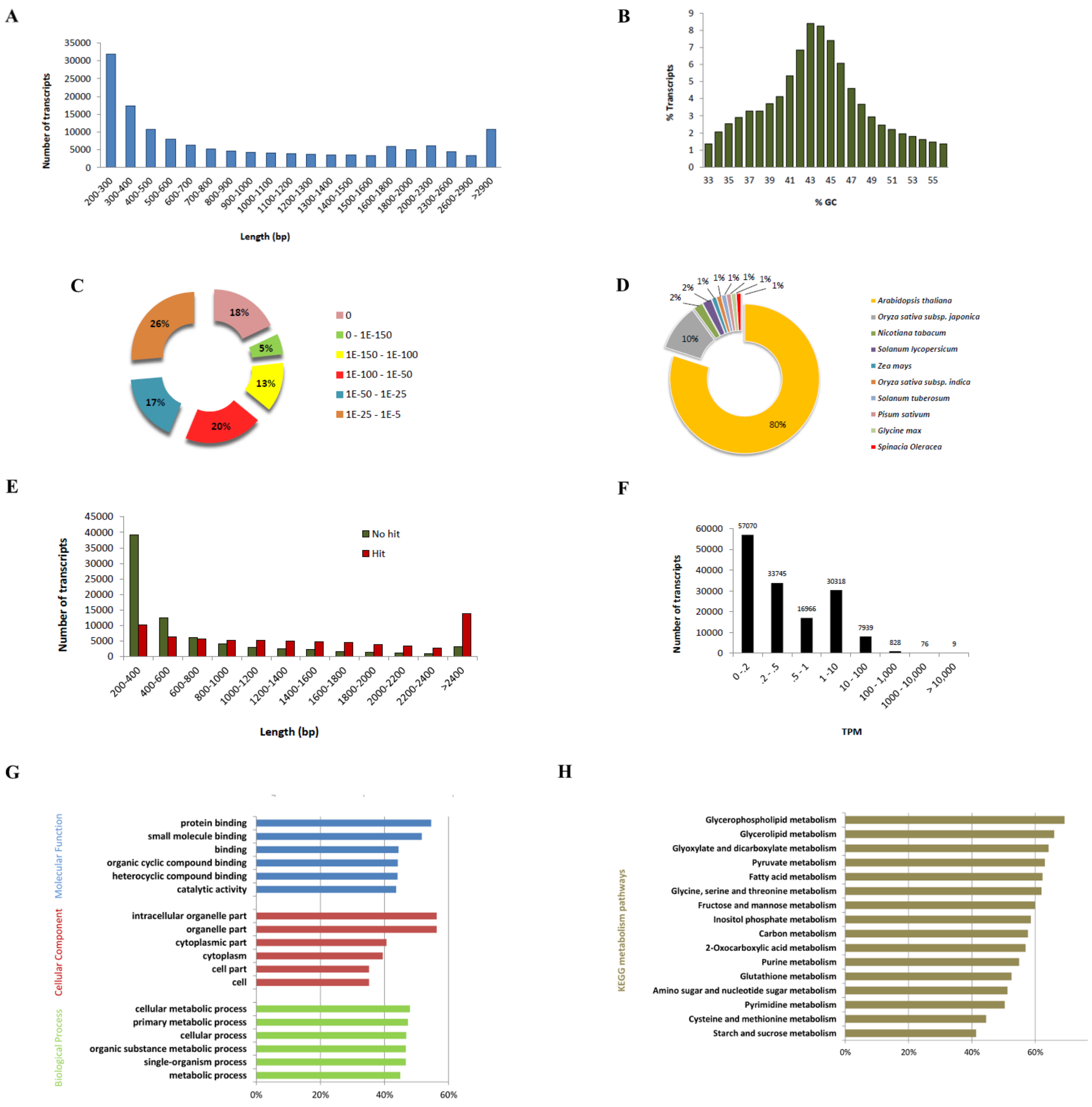

Supplementary Figure S1. Summary of assembled transcripts and annotation. **(A)** Transcript length distribution. **(B)** GC content distribution of transcripts. **(C)** E-value distribution of best hits. **(D)** Species distribution of homologous transcripts. **(E)** Transcript length distribution of transcripts with and without annotation. **(F)** Average transcript expression distribution (transcripts per million reads) among chia accessions. **(G)** Gene ontology classification and **(H)** KEGG metabolism pathways enrichment analysis.

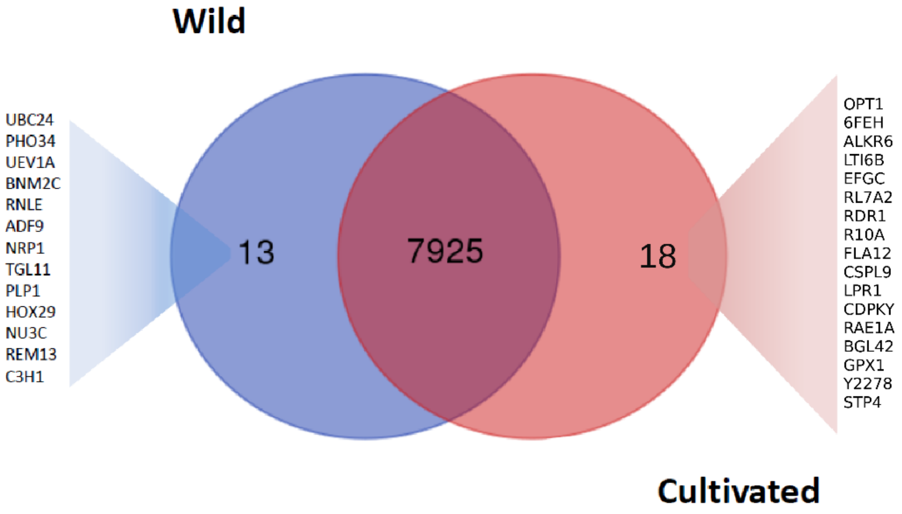

Supplementary Figure S2. Venn diagram showing the number of identified genes shared or exclusive between cultivated and wild accessions.

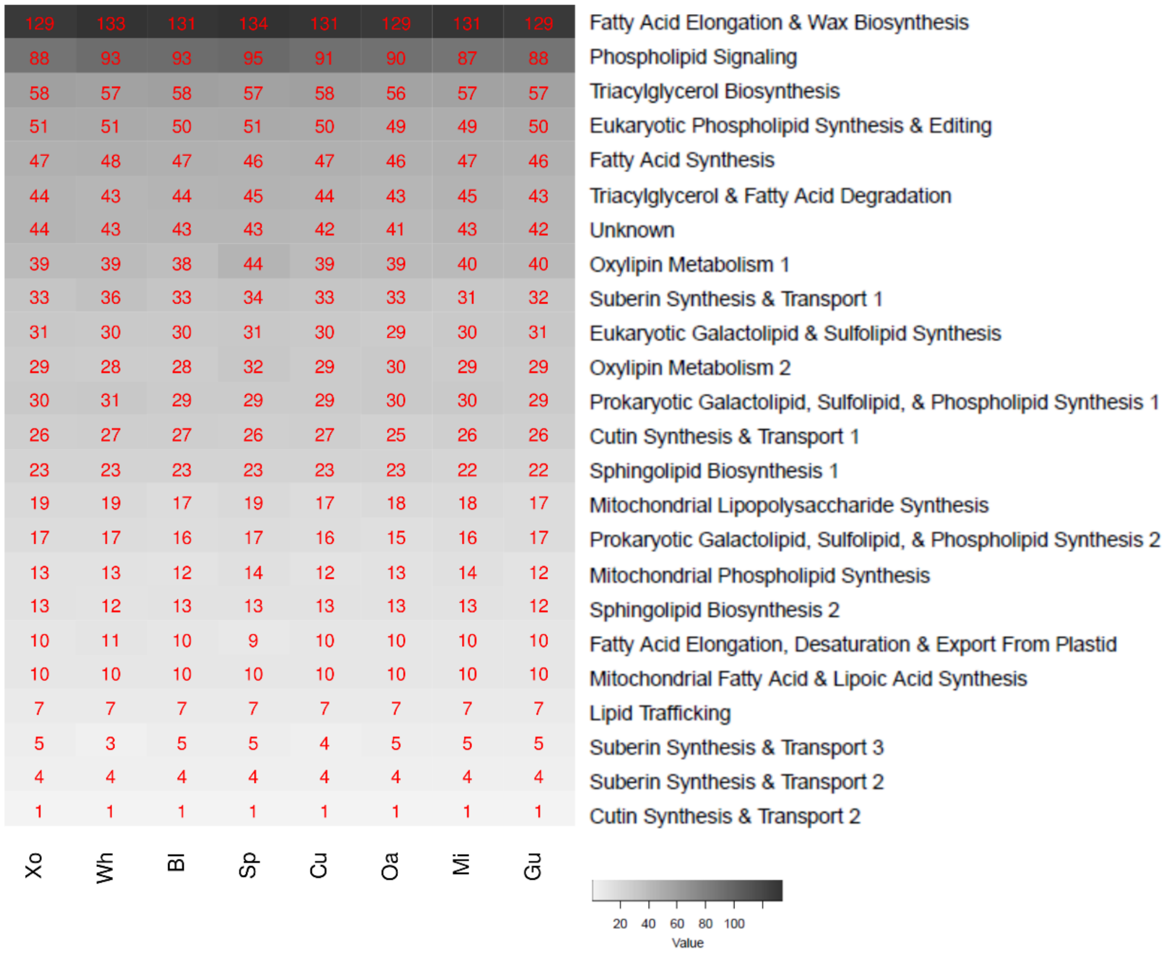

Supplementary Figure S3. Heatmap of the number of categorized lipid-related genes detected in the different accessions according to the ARALIP database.

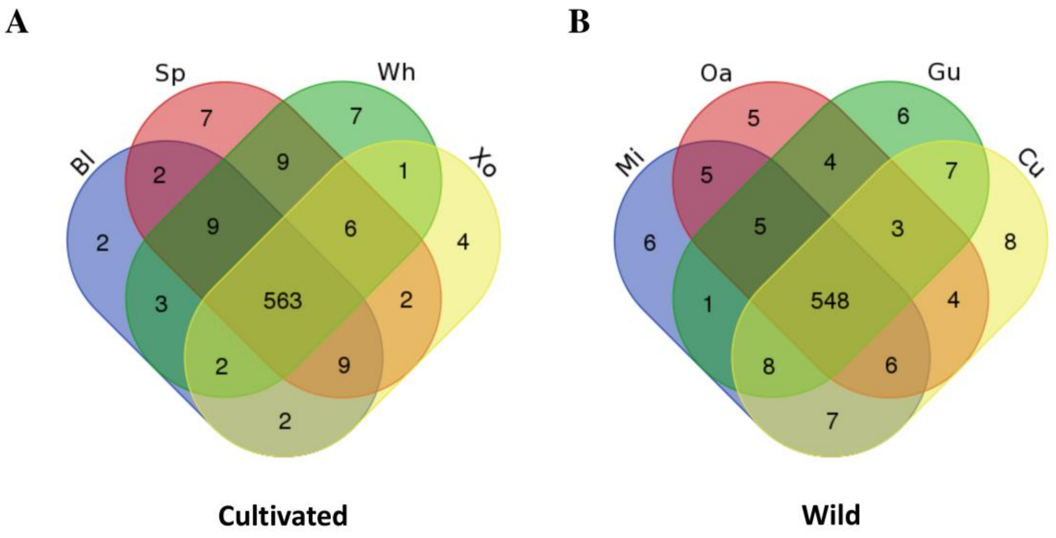

Supplementary Figure S4. Venn diagram showing the number of lipid-related genes shared or exclusive within the cultivated **(A)** and wild **(B)** accessions.

Supplementary Table S1. Summary of transcriptome *de novo* assembly.

Total genes:	83,409
Total transcripts:	146,951
GC content (%):	45.4
Minimum contig length (bp):	201
Maximum contig length (bp):	66,672
Median contig length (bp):	687
Contig N50 (bp):	1,949
Total assembled bases:	171,001,990
